# Atopic dermatitis: a need to define the disease activity

**DOI:** 10.3389/fmed.2023.1293185

**Published:** 2023-11-03

**Authors:** Axel De Greef, Laurence de Montjoye, Thomas Bieber, Marie Baeck

**Affiliations:** ^1^Department of Dermatology, Cliniques universitaires Saint-Luc, Université catholique de Louvain (UCLouvain), Brussels, Belgium; ^2^Christine Kühne-Center for Allergy Research and Education, Davos, Switzerland

**Keywords:** atopic dermatitis, terminology, consensus, disease activity, disease modification, disease modification treatments, biomarkers

## Introduction

Atopic dermatitis (AD) is one of the most frequent inflammatory skin diseases characterized by flares and remissions of eczematous lesions and intense itching (1).

Mild disease is the most common severity presentation, nevertheless it is estimated that 20–30% of patients suffer from moderate-to-severe AD (2). Until recently, there was an unmet need for long-term disease control in these more severe patients. The emergence of new systemic therapies has led to significant clinical improvement for many patients. Therefore, during the long-term management of AD, it is important to properly characterize the severity of the disease in order to allow optimal and individual therapeutic decisions. However, the terminology used needs to be clearly defined.

## Discussion

There is still some confusion between the concepts of severity, activity (clinical and/or biological), long-term disease control, and short- or long-term remission of AD. Several scores have been developed to evaluate the severity of the disease (3), each of these scores assessing either objective signs (type and extent of skin lesions), subjective symptoms (pruritus, pain, sleep disturbances), quality of life or disease control. Besides the fact that they must often be combined to account for all aspects of the disease burden, all these scores only allow the evaluation of the disease at a certain time (mainly at consultation) or over a maximum of 7 days (with the RECAP and ADCT scores). They do not take into account the *clinical activity* which could be defined as the fluctuating course of AD signs and symptoms over several weeks/months, experienced by most of the patients, regardless of the disease severity (4). Indeed, AD severity may fluctuate considerably over time, even with the (new) standard of care treatments (4, 5). By repeating “static” scores, the severity of AD and/or impact on quality of life of patients with such fluctuating disease, may be underestimated. A “dynamic” evaluation needs to be developed in order to determine the longitudinal phenotype of the patient, i.e., (i) controlled AD patients; (ii) non-controlled AD patients that presented with fluctuating disease and high activity/variability; (iii) non-controlled AD patients that presented with non-fluctuating, continuously severe disease.

Furthermore, the suggested “disease-modifying” potential of biologics, such as with anti-interleukin-4 receptor α (IL-4Rα) biologics, introduces the possibility of long-term remission of AD after treatment discontinuation. “Long-term remission” refers to “a state of absence of disease activity that lasts for an extended period, usually at least 1 year” (6). It would imply a state where both objective and subjective clinical scores remain low (even zero) over time. The possibility of such drugs to impact on the atopic and non-atopic comorbidities of AD is also discussed (6).

In addition to clinical parameters, there now exists a notion of disease *activity* on a *biological level*, which could become a future important factor in patients' evaluation, in decision-making and in assessing whether or not to maintain treatments over the long term (6). As AD in known to lead to systemic inflammatory state (7, 8), the presence or absence of subclinical inflammation could be a relevant marker. Persistence of pathogenic skin-resident memory T cells in nonlesional AD skin 4 months after effective anti-inflammatory treatment, suggest that immune memory might be involved in AD relapses (9, 10). Additionally, a decrease or loss of function of regulatory T cells, has been hypothesized to activate Th2 cells, leading to the development and maintenance of AD (11). Furthermore, autoimmunity (immunity directed against keratinocytes-derived proteins) is assumed to be involved in the pathophysiology of AD (12). Detection of autoantibodies IgE has been associated with presence of Th2 comorbidities (13) but the direct link with AD severity is still unclear. Cytokines produced by autoreactive T-cells could also directly exacerbate the skin lesions (14). Studies which tend to characterize patients' immunologic signature (endotype) could allow the advent of biomarkers predictive of the therapeutic response and biomarkers prognostic for the disease progression (15).

In conclusion, the effective management of AD patient must be based on valid and reliable outcomes and requires not only an assessment of the severity, but also of disease activity on a clinical level but also on the “invisible” biological one. There is a need for clinical scoring that effectively assesses the “dynamic” aspect of the disease, over time. In addition, the notion of AD biological activity requires further investigations.

## Author contributions

AD: Conceptualization, Writing—original draft. LM: Conceptualization, Supervision, Validation, Writing—review & editing. TB: Conceptualization, Supervision, Validation, Writing—review & editing. MB: Conceptualization, Supervision, Validation, Writing—review & editing.
